# Dropout at Danish vocational schools: does the school’s health promotion capacity play a role? A survey- and register-based prospective study

**DOI:** 10.1186/s12889-020-08955-4

**Published:** 2020-05-26

**Authors:** Maja Thøgersen, Mette Aadahl, Peter Elsborg, Charlotte Demant Klinker

**Affiliations:** 1 Health Promotion, Steno Diabetes Center Copenhagen, Niels Steensens vej 6, 2820 Gentofte, Denmark; 2 Centre for Clinical Research and Prevention, Bispebjerg and Frederiksberg Hospital, Nordre Fasanvej 57, Hovedvejen 5, 2000 Frederiksberg, Denmark

**Keywords:** Vocational education and training, Student dropout rates, Health promotion capacity, Prevention, Organizational structure

## Abstract

**Background:**

School dropout rates and risky health behavior is common among students in vocational education and training (VET) schools. Students with poor physical and mental health are more likely to drop out, and as such VET schools may be an important setting for health promotion initiatives, not only to support a healthy lifestyle, but also to assure completion of education. A common feature of successful health promotion at VET schools is a high health promotion capacity at the school level. This study aimed to investigate the association between VET school’s health promotion capacity and later student dropout rates. Secondary, we explored other school characteristics associated with student dropout rates.

**Methods:**

This prospective study comprised 58 Danish VET schools offering basic programs. Health promotion capacity was assessed using questionnaire data from 2017 from school managers and teachers, and this was combined with register-based data on student dropouts the following year. Health promotion capacity was assessed using six scales, representing six underlying domains, and managers and teachers’ ratings of these were compared using t-test. Associations between health promotion capacity and student dropout rates as well as associations between school characteristics and student dropout rates were analyzed using multiple linear regression.

**Results:**

No associations between VET schools’ health promotion capacity and student dropout rates were observed, neither for the schools’ overall health promotion capacity or for any of the six underlying domains (*p* = 0.17–0.84). School managers assessed health promotion capacity significantly higher than teachers overall and within all domains (*p* < 0.05). Moreover, student dropout rates were significantly lower at schools with a higher proportion of ethnic Danish students, VET-students at higher educational level and schools located in the Western part of Denmark (*p* < 0.05).

**Conclusion:**

No associations between VET schools’ health promotion capacity and student dropout rates were observed. This may be due to a relatively short follow-up time in our study and future research may reveal if VET school health promotion capacity may affect dropout rates over a longer time period. Moreover, more work is needed to further develop instruments for measuring health promotion capacity in a VET school context as well as other contexts.

## Background

A high school dropout rate and risky health behavior is common among students in vocational education and training (VET) schools in both Denmark and other European countries [1–4]. VET is a practically oriented secondary education, and compared to high schools, VET students have a more pronounced risk behavior in terms of poor diet, frequent smoking and low levels of physical activity [1]. VET schools are characterized by having a high proportion of students with a low socioeconomic background. Education is a key determinant for health and longevity, as lower educational level is associated with poorer health and shorter life expectancy [5, 6]. In Denmark, about 19% of a youth cohort starts a VET program, however, only 50% of the students complete this education [2]. This may lead to poorer job prospects and increased health inequity [2, 7, 8].

Although student dropout rates are often characterized by complex and individual determinants [8, 9], research emphasizes that a school’s social and organizational environment has beneficial effects on school completion [10, 11]. Furthermore, students with poor physical and mental health are more likely to drop out [8, 12]. Since adolescence is a critical period in developing sound behavioral patterns into adulthood [13–15], and as school represents a major part of the students’ daily life, it may be an important setting for health promotion initiatives [10, 16–18], not only to support a healthy lifestyle, but also to assure completion of education. Following this, the Danish government introduced the VET-reform in 2015, aiming to promote health and reduce school dropout [2].

Evidence of associations between schools’ health promotion initiatives, such as tobacco prevention or increase of social cohesion and physical activity, and student dropout rates is sparse [5]. One study showed that an intervention targeting social relations and tobacco prevention reduced student dropout rates significantly through increased school connectedness in the intervention group compared to a matched control group [19]. Further, case studies indicate that physical activity interventions have the potential to increase student wellbeing and strengthen social relations among VET students and teachers, thereby reducing dropout rates [20–22]. A common feature of these studies is the presence of a high health promotion capacity at the school level, covering motivated teachers and school managers and proper organizational support structures including sufficient resources and focused implementation strategies [20–22].

WHO defines health promotion capacity building as ‘*the development of knowledge, skills, commitment, structures, systems and leadership to enable effective health promotion’* [23]*.* When using the concept *health promotion capacity* in our study, we rely on WHO’s definition as well as the conceptual framework by Aluttis et al. [24], including domains of organizational development, financial and human resource allocation and leadership commitment. A high health promotion capacity also predicts a more successful implementation of an intervention [25, 26]. WHO proposed, that building capacity for health promotion requires institutionalizing it, hereby integrating health promotion into blocks of financial and human resources, knowledge management, supportive leadership and capacity for effective implementation [27]. As such, it is likely that a school’s engagement and capacity to conduct health promotion initiatives is associated with student dropout rates.

To the best of our knowledge, no studies have previously investigated whether VET schools’ capacity in health promotion initiatives affect student dropout rates. A potential association may underline the importance of health promotion activities being conducted in this setting and help schools prioritize their preventive efforts, when aiming to avoid student dropout.

The primary aim of this study was to investigate whether VET schools’ health promotion capacity, measured by six underlying domains (*knowledge development, communication, resources, school-based leadership, teaching staff* and *students*), is associated with student dropout rates within the first six months of education. Second, to examine if other school characteristics (geographical location, school type, school size, VET educational level and students’ age and ethnicity) can explain dropout rates.

## Methods

### Setting

In the Danish educational system, most young people (4 out of 5) between the age of 15–17 years old choose to continue from compulsory school into either academically oriented upper secondary schools (approximately 3/5) or VET schools (1/5). VET is a practically oriented education, preparing students for immediate entry into the labor market within e.g. agricultural, commercial, technical or social healthcare professions [2]. The training includes a 6–12 months school-based basic program, followed by the main program, alternating between school and apprenticeship. Approximately 20% of the students combine their normal VET-level with an academical upper secondary education, giving further access to higher education (VET-higher). Most of the student dropouts occur during the basic program and is generally lower among VET-higher students [28]. The average age of students enrolling the basic program is 21.5, as some students also enroll a VET program later in adult life [29].

In 2015, the Danish government introduced an educational reform targeting higher completion of the VET program [2]. A cornerstone of the reform was to create more attractive school environments. More specifically the schools were to improve students’ health and wellbeing by increasing social cohesion between students and by implementing 45-min of physical activity per day. Such initiatives were hypothesized to promote health and support motivation and learning and thus reduce dropout rates [2]. The reform was to be implemented in the first part of VET, the basic program (6–12 months), as this is where student dropout is particularly high [2].

### Design

This study was conducted at school level and employed a prospective design, linking VET schools’ health promotion capacity, measured in spring 2017, with the schools’ dropout rates at the basic course level one year subsequently. The VET schools’ dropout rates among students and information on school characteristics were obtained from the official register of the Danish Ministry of Education [30], providing statistics on all Danish VET schools. Data on health promotion capacity were obtained from a self-administered national survey, that aimed to map Danish VET schools’ health promotion initiatives and implementation capacity [31].

**Fig. 1:**
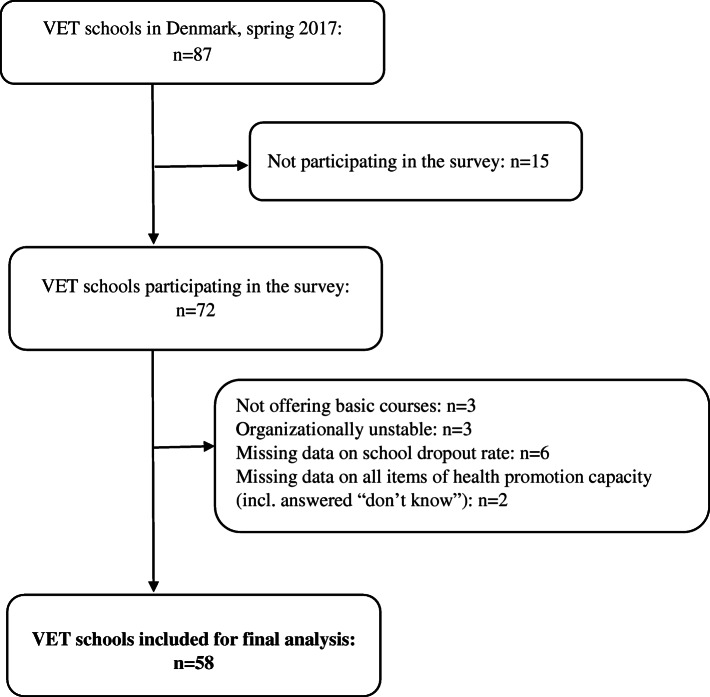
Flow diagram for VET schools included in the study

The questionnaire was originally distributed to all 87 Danish VET schools and asked to be completed by all school managers. The school managers were then told to ask a sample of teachers to complete it. Measures were taken to minimize selection bias including a written explanation on selecting teachers that would constitute a representative sample of teachers, e.g. that they should choose teachers that represented different educations, differences in teaching experience and attitudes towards health promotion. A total of 72 schools took part in the survey (response rate 82%), informed by 192 school managers and 378 teachers. We excluded VET schools that did not provide basic courses (*n* = 3), that were organizationally unstable through 2017 (e.g. three schools merging into one) (*n* = 3), with missing data on the schools’ dropout rates (*n* = 6), and schools with missing data on all items of health promotion capacity (*n* = 2), resulting in 58 eligible VET school for final analyses, informed by 313 respondents (125 school managers and 188 teachers) (Fig. 1). The population used for non-response analyses was 82 schools, as information used in the analyses was not available in five schools. The individual responses from managers and teachers respectively were aggregated to the school level.

### Outcome measures

The VET schools’ student dropout rates were defined as the proportion of students, who started a basic program in 2017, and dropped out within six months later, without choosing another VET subject area. Data was derived from the official register of the Danish Ministry of Education at May 21st, 2018 [30].

### Exposure variables

Our main exposure was VET schools’ *health promotion capacity*. We categorized health promotion capacity into six domains, inspired by the framework of Aluttis et al. [24]: *Knowledge development, communication, resources, school-based leadership, teaching staff* and *students* (Table 1). Each domain included 2–5 items on a 5-point Likert scale (where 1 is ‘to a very low degree’ and 5 is ‘to a very high degree’). All items also included a “don’t know” option as this was a plausible answer for some items. First, the domain score for each respondent was calculated as the mean score of the individual responses, excluding items where the respondent had answered “don’t know” or with missing data. Then the domain score for each VET-school was aggregated as the mean score of the individual responses from managers and teachers respectively.

**Table 1:** Domains and content of a questionnaire measuring health promotion capacity

Health promotion capacity domain	No. of items included in final model	Key Content	Example of wording of item
**Knowledge development**	3	• Systematic use of evidence-based knowledge	*The school’s work on health promotion is based on a systematic use of knowledge*
		• Collaboration takes place across the organization	
		• Network of engaged employees is established	
**Communication**	3	• External communication on health promotion efforts	*The school management has made efforts to create awareness on the school’s work on health promoting to the surrounding world*
		• Daily focus on health promotion	
		• Increased focus on health promotion after the VET reform	
**Resources**	5	• Not enough time to work with health promotion	*The school’s employees have enough time to work with health promotion*
		• Enough staff has time allocated	
		• Enough finances are allocated	
		• Health promotion is not prioritized	
		• The school has an overview over possible external stakeholders	
**School-based leadership**	5	• Support from management	*The management support the employees’ work on health promotion*
		• Clear goals and directions	
		• Management believes health promotion is important for student motivation and learning	
		• Assigned leaders advocate for health promotion	
		• Health promotion activities are initiated by leaders	
**Teaching staff**	2	• Employees are involved	*Employees are involved in development of or decisions on health promotion activities*
		• Employees have influence	
**Students**	2	• Students are involved	*Students are involved in development of or decisions on health promotion activities*
		• Students have opportunities to improve own health during school	

The questions were developed based on current literature [26, 32, 33] and piloted in the target- and a reference group (people with expertise on the VET school context and health promotion questionnaires in general), aiming for a high content- and face validity. Based on the pilot, minor adjustments were made, as described elsewhere [34]. Moreover, a sub-analysis of the present study tested the construct validity and internal consistency by confirmatory factor analysis and Cronbach’s alpha tests respectively (Additional file 1).

### Covariates

To assess the association between school characteristics and student dropout rates and to adjust for possible confounders, we included the following covariates: *Geographical location*, as previous research had found it to be associated with student dropout [35]. It was dichotomized into schools located in the Eastern part of Denmark (Capital Region of Denmark and Region Zealand) and the Western part of Denmark (Region of North-, Central- and Southern Denmark). *School type* was categorized according to the school’s main subject area: “Food, agriculture, hospitality” (Food), “Care, health, pedagogy” (Care), “Administration, commerce, business” (Business) and “Technology, construction, transportation and combination” (Tech and comb.) schools. “Combination” schools cover more than one main subject area and were merged with “Tech” schools, as all “Combination” schools provided “Tech” and only four schools offered “Tech” alone. *VET-level* was measured as the school’s percentage of VET-higher students, who combined their normal basic program with subjects at upper secondary level. As both the school type and VET-level are new structures as a result of the reform [2], their effect on student dropout rates seem unexplored. *School size* was measured as the number of students, who enrolled a basic program in 2017. Associations on school size and student dropout rates seem inconsistent in existing literature [36, 37]. *Student age* was measured as the school’s average age among students, who started at the basic program in 2017. Both increasing age and ethnicity are found to be associated with dropout among Danish VET students [8]. *Danish ethnicity* was measured as the school’s percentage of students of Danish origin, who started at the basic program in 2017. Thus, the remaining proportion of students are immigrants or descendants of immigrants. All covariates were derived from the register of the Danish Ministry of Education on 21st May 2018 [30].

### Statistical analyses

To test the construct validity and internal consistency of the questionnaire, three different factor structures of the health promotion capacity questions were tested with confirmatory factor analysis, performed in the R package Lavaan [38] and with Cronbach’s alpha test using SPSS version 24.

In all main analyses, all data on respondent level was aggregated to the school level to account for clustering of responses from the same school. Initially we performed a Pearson correlation analysis of the covariates and tested for multicollinearity. Non-response analyses were performed using Chi-square and independent sample t-test, comparing participating and non-participating schools based on school characteristic variables.

Differences in health promotion capacity between school managers and teachers were tested using independent sample t-test.

Associations between VET schools’ health promotion capacity and student dropout rates were analyzed by linear regression for each of the six domains of health promotion capacity and for the total health promotion capacity score (crude associations, model 1). Using multiple linear regression, we adjusted for geographical location, school type, school size, VET-level, student age and ethnicity (model 2). Associations between school characteristics and student dropout rates were derived from the regression models based on total health promotion capacity. Normality, linearity and homoscedasticity in all regression models were tested and confirmed.

A 5% statistical significance *p*-value was applied. Analyses were performed using SPSS version 24.

### Ethical considerations

Information about confidentiality and voluntary participation were provided in adherence to the Danish Data Protection Legislation [39]. The data used in our study did not involve personal or sensitive data: thus, as this is not required for social science in Denmark [40, 41], no institutional ethical- or data approval was obtained.

## Results

### Validity and reliability of the questionnaire

Based on the confirmatory factor analysis, the final factor structure showed a borderline acceptable model fit according to reference values [42] on the following model fit indices CFI = 0.90, TLI = 0.87 and RMSEA = 0.07 (95% CI: 0.07–0.08) and with all factor loadings above 0.4 (*p* < 0.00). Based on reference thresholds [43], all six domains showed acceptable alpha values ranging from α = 0.6–0.8 (Additional file 1).

### Participating schools

Of the 58 included VET-schools, the number of respondents per school before aggregating data into the school level varied from 1 to 35 respondents. This included 1–7 school managers (median: 2.0) and 0–28 teachers (median: 2.0) per school.

A non-response analysis showed no difference between participating and non-participating schools based on geographical location, school size, school type, proportion of VET-higher students and students of Danish ethnicity (*p* > 0.05). However, the included schools had a significantly higher average student age (22.1 years, SD:2.6) compared to the non-participating schools (21.7 years, SD:2.8) (*p* = 0.01).

Table 2 shows characteristics of the included VET schools. Considerable variation was seen in geographical location, school type and -size, proportions of VET-higher students and ethnicity. Correlations between school characteristics ranged from (+/−) 0.0–0.6. Additionally, there was no indication of multicollinearity according to threshold values [44], as variance inflation factor ranged from 1.1–1.9 and tolerance from 0.5–0.9.

**Table 2:** Characteristics of VET schools (*n* = 58)

	*n* (%)	Mean (SD ^a^)	Range
**Geographical location**			
West (Region of North-, Central- and Southern Denmark)	43 (74.1)	–	
East (Capital Region of Denmark and Region Zealand)	15 (25.9)	–	
**School type**			
Care, health, pedagogy	14 (24.1)	–	
Technology, construction, transportation and combination ^b^	23 (39.7)	–	
Food, agriculture, hospitality	6 (10.3)	–	
Administration, commerce, business	15 (25.9)	–	
**School size**^**c**^	–	624 (581)	36–2775
**VET-higher level, %**	–	14.8 (15.0)	0.0–82.0
**Student age**^**d**^	–	22 (2.6)	17–28
**Danish ethnicity, %**	–	84.3 (10.5)	53.7–99.1
**Student dropout rate**^**e**^**, %**	–	17.4 (5.7)	6.1–34.0

^a^*SD* standard deviation

^b^ Combination schools: schools providing more than one main subject area. All combination schools provide trainings within the main area “Technology, construction and transportation”

^c^ School size: number of students, who enrolled a basic program in 2017

^d^ Student age: mean age of students at the school enrolled in 2017

^e^ Student dropout rate: proportion of students, who enrolled a basic program in 2017, and dropped out within six months, without transferring to another VET subject area

### Health promotion capacity

In general, VET schools’ health promotion capacity was scored significantly higher by school managers than by teachers, both in terms of the total capacity score and within the six underlying domains (*p* < 0.01) (Table 3). Both school managers and teachers scored *teaching staff* to be highest, whereas the domains of *resources* and *knowledge development* scored lowest. The proportion of respondents who provided data on more than 2/3 of items of the domain varied from 92.3% in *communication* (78.3% with no missing and 14.1% with 1 missing item) to 78.6% in *resources* (61.7% with no missing and 16.9% with 1 missing item). Within all domains, missing data was more prominent among teachers than school managers (Additional file 2).

**Table 3:** VET schools’ health promotion capacity, rated by school managers (*n* = 58 schools) and teachers (*n* = 38 schools)

	School managers score (*n* = 58)	Teachers score (*n* = 38)	
	Mean (SD)	Mean (SD)	*p*-value
**Health promotion capacity domain**			
Knowledge development	3.0 (0.8)	2.6 (0.8)	*0.01*
Communication	3.1 (0.5)	2.6 (0.6)	*0.00*
Resources	3.0 (0.6)	2.5 (0.6)	*0.01*
School-based leadership	3.5 (0.7)	2.9 (0.8)	*0.00*
Teaching staff	4.1 (0.6)	3.6 (0.7)	*0.00*
Students	3.2 (0.7)	2.8 (0.5)	*0.00*
**Total health promotion capacity**	3.3 (0.5)	2.8 (0.5)	*0.00*

### Associations between health promotion capacity and student dropout rates

There were no significant associations between VET schools’ health promotion capacity, scored by school managers or teachers, and student dropout rates (Tables 4 and 5). No associations were found for the total school capacity or for any of the six underlying domains, neither in the crude models nor in the adjusted models. Inspection of r^2^ values revealed that school capacity explained 0–6% of the student dropout rates, while the models including covariates explained 47–61% of the variation in student dropout rates.

**Table 4:** Impact of VET schools’ health promotion capacity, scored by school managers, on student dropout rates (*n* = 58 schools)

	Model 1 (crude)			Model 2 (adjusted ^b^)		
	β estimate ^a^	95% CI	*p*-value	β estimate ^a^	95% CI	*p*-value
**Health promotion capacity domain**						
Knowledge development	1.39	−0.37;3.16	*0.12*	0.95	−1.01;2.91	*0.34*
Communication	2.20	−0.55;4.94	*0.11*	0.96	−1.68;3.59	*0.47*
Resources	0.06	−2.59;2.70	*0.97*	−1.28	−3.81;1.24	*0.31*
School-based leadership	1.11	−1.06;3.28	*0.31*	0.76	−1.43;2.95	*0.49*
Teaching staff	−1.23	− 3.70;1.25	*0.33*	0.03	−2.21;2.26	*0.98*
Students	−0.05	−2.19;2.09	*0.96*	−0.25	−2.21;1.71	*0.80*
**Total health promotion capacity**	0.00	−2.17;4.15	*0.53*	0.30	−2.95;3.55	*0.85*

^a^ Estimates derived from student dropout rates as dependent variable, giving the percentage change in student dropout rate per one increase in health promotion capacity on a 5-point Likert scale (from “very low degree” to “very high degree”)

^b^ Adjusted for geographical location, school size, school type, VET-level, students’ age and students’ ethnicity

**Table 5:** Impact of VET schools’ health promotion capacity, scored by teachers, on student dropout rates (*n* = 38 schools)

	Model 1 (crude)			Model 2 (adjusted ^b^)		
	β estimate ^a^	95% CI	*p*-value	β estimate ^a^	95% CI	*p*-value
**Health promotion capacity domain**						
Knowledge development	0.57	−1.93;3.08	*0.65*	−0.38	−2.67;1.92	*0.74*
Communication	2.53	−0.84;5.90	*0.14*	1.00	−2.83;4.82	*0.60*
Resources	−1.64	−5.00;1.72	*0.33*	−0.84	−4.32;2.65	*0.62*
School-based leadership	−0.43	−3.21;2.36	*0.76*	−0.84	− 3.31;1.64	*0.49*
Teaching staff	0.67	−2.51;3.86	*0.67*	1.23	−2.16;4.62	*0.46*
Students	2.75	−1.58;7.08	*0.21*	0.21	−3.96;4.37	*0.92*
**Total health promotion capacity**	0.70	−3.81;5.20	*0.76*	−0.82	−5.37;3.72	*0.71*

^a^ Estimates derived from student dropout rates as dependent variable, giving the percentage change in student dropout rate per one increase in health promotion capacity on a 5-point Likert scale (from “very low degree” to “very high degree”)

^b^ Adjusted for geographical location, school size, school type, VET-level, students’ age and students’ ethnicity

### School characteristics and student dropout rates

The adjusted regression model based on managers’ scores on total health promotion capacity revealed, that student dropout rates were significantly lower at schools with a higher proportion of VET-higher students (*p* = 0.01), with a higher proportion of ethnic Danish students enrolled at a basic program (*p* = 0.04), and in schools located in the Western part of Denmark (*p* = 0.04) (Table 6). School type, school size and students’ average age were not associated with student dropout rates. A sensitivity analysis based on the teacher score or applying any of the underlying six domains of health promotion capacity did only produce minor changes in the estimates and no significant changes in these results.

**Table 6:** Impact of VET school characteristics on student dropout rates (*n* = 58)

	Crude			Adjusted ^e^		
	β estimate	95% CI	*p*-value	β estimate	95% CI	*p*-value
**VET-level**	−0.18	−0.27; − 0.10	*0.00*	−0.23	− 0.39; − 0.07	*0.01*
**Geographical location**^a^	−3.38	−6.70; − 0.06	*0.05*	−6.22	−12.01; − 0.43	*0.04*
**Danish ethnicity**	− 0.24	−0,37; − 0.11	*0.00*	−0.20	− 0.39; − 0.01	*0.04*
**School size**^b^	0.00	0.00; 0.01	*0.07*	−0.00	−0.01; 0.00	*0.45*
**Student age**^**c**^	0.93	0.39; 1.47	*0.00*	−0.04	−1.09; 1.02	*0.95*
**School type**^d^						
Food, agriculture and hospitality	−3.32	−8.28; 1.64	*0.19*	−2.55	−14.28; 9.18	*0.66*
Care, health, pedagogy	2.05	−1.62; 5.72	*0.27*	−9.59	−19.90; 0.73	*0.07*
Administration, commerce, business	−3.36	−6.95; 0.23	*0.07*	−4.30	−11.92; 3.33	*0.26*

^a^ Schools located in the western part of Denmark compared to Eastern reference schools

^b^ Number of students, who enrolled a basic program in 2017

^c^ Mean age of students at the school enrolled in 2017

^d^ Schools providing any of the three main subject areas ‘Food’, ‘Care’ or ‘Business’ compared to the reference group ‘Tech and comb.’

^e^ Adjusted for VET-level, geographical location, students’ ethnicity, school size, students’ age, school type and total health promotion capacity score based on managers’ scores

## Discussion

We found no association between VET schools’ health promotion capacity and student dropout rates, neither for the overall health promotion capacity or for the six underlying domains; *knowledge development, communication, resources, school-based leadership, teaching staff or students*. School managers consistently scored health promotion capacity higher than teachers. We found a lower student dropout rate at schools with more VET-higher students, more students of Danish ethnicity and at schools located in the Western part of Denmark compared to East. School size, school type and students’ age did not affect student dropout rates in this study. The instrument used to measure health promotion capacity had a reasonable model fit and have the potential to eventually be used in other population-based studies, although the development of the questionnaire remains a work in progress.

Health promotion interventions have previously been found to lower student dropout rates at VET schools in three studies [19, 20, 22]; all schools characterized by a high level of health promotion capacity in terms of human and material resources. However, we find that our study differed on a number of parameters. First, as our study did not focus on a specific intervention, but on general health promotion capacity, we do not know the actual level of health promotion activities present at the schools. Though Heinze and colleagues revealed, from the same survey data, that VET schools with a high health promotion capacity had more proactive attitudes towards smoke-free school-hours [31], further studies should explore if health promotion activities are more effective at high capacity schools, and how this may influence student dropout rates. Secondly, we did not include a measure on social relations and social connectedness among students. Andersen et al. [19] and Ingholt et al. [16] illustrated, that social cohesion was a strong mediator when reducing student dropout rates through a smoking prevention intervention. Social relations has further been highlighted as important in an intervention aiming to lower student dropout through physical activity [22]. As social cohesion and educational completion are highly correlated, it may require more attention, when planning health promotion interventions. Further development in the field of measuring health promotion capacity at the school level may also need to include measures to account for more social indicators at the student level.

Implementation processes are both time- and resource consuming and often requires long implementation time before any effects may be seen [45]. The Danish VET-reform was introduced in August 2015, following high demands for changes in organizational structure and staff and students’ daily practice. First of all, following a new reform, goal planning and strategies are expected to be more prominent among managers, and only later among teachers (even later among students) [34]. This may explain why health promotion capacity was rated higher by school managers than by teachers in our study. However, this difference was also expected as a natural tendency when comparing attitudes at management- and employee level [46]. Secondly, Bosworth et al. [45] conclude, that school-based health promotion interventions require at least a 3 year period to be fully implemented, while Andersen et al. [19] employed a 2 year follow-up period, when showing an effect of tobacco prevention on student dropout rates. Hence, a longer follow-up time than our one-year period may be needed to conclude, if health promotion capacity affects VET student dropout rates.

Surprisingly, we did not find an impact of the two domains, *students* and *teaching staff* on student dropout rates. Involvement of target groups in health promotion initiatives is found to increase motivation and participation, thereby strengthening implementation [26, 47, 48] and lower dropout rates [19, 22]. Moreover, a field study by Grønborg [49] revealed that VET students had high absence in school and refused to participate in sport activities, mainly due to lack of influence and involvement. Our results should be interpreted while keeping in mind, that we did not assess students’ opinion of being involved, but managers and teachers’ opinion only. As staff and students may not share the same attitudes [50], future studies should also assess the students’ opinion of being involved.

Our results on a lower student dropout rate at schools with a higher proportion of ethnic Danish students are mirrored by other studies [8, 19], highlighting that in the development of health promotion initiatives, efforts must be done to adapt the initiatives specifically to this target group [49], e.g. by including students with a different ethnic and cultural background in the intervention development. The low student dropout rates in Western schools compared to Eastern schools has previously been explained by traditions of educational completion, fewer educational alternatives due to smaller cities in the Western region compared to the Eastern region with larger cities, and that closure-threatened schools in the Western region prioritize retention over academic results [35]. Our findings should therefore be a point for political attention. Moreover, Rumberger [10] states, that student dropout may be associated with school size, but that inconsistent findings may be explained by a non-linear relationship, where medium-sized school seem more effective than small and large schools. We are however not able to confirm this hypothesis in our study.

This is the first study to investigate the population of VET-higher students after the reform was brought into effect in 2015. We found a lower dropout rate at schools with more VET-higher students indicating that a social gradient has been introduced within the VET-school program. VET-higher education programs provide higher educational opportunities [2] and may motivate educational completion. Also, students choosing a higher educational level often have a stronger socioeconomic background and a lower dropout rate than students enrolling in a normal VET program [9], which may explain this finding.

### Strengths and limitations

This is, to our knowledge, the first study to apply a health promotion capacity questionnaire among VET schools and to study its impact on student dropout rates, which is a strength given the focus of health promotion in the Danish VET reform [2] and in the international context more generally [5]. The opportunity to use a nationwide survey sample and combine it with reliable register-based data on student dropout rates made it possible to build a longitudinal dataset with inclusion of the entire population, and minimize information bias in terms of misclassification and recall discrepancy [51]. A longer timeframe may however be needed as the schools are still implementing the VET health promoting aspects of the VET reform, and this will become possible to employ in the future years, as new student dropout rates for the VET schools will be updated.

Health promotion capacity was operationalized based on an existing theoretical model, developed in dialogue with an expert reference group and pilot tested in the target group to strengthen both face-, content- and conceptual validity [34]. By testing the construct validity and internal consistency by factor analysis and Chronbach’s Alpha test, the tool was considered both acceptable and a valuable first tool for measuring health promotion capacity in VET schools. Further development and qualified validation of the health promotion capacity instrument is still recommended, as the lack of association may also reflect the ability of the instrument to measure health promotion capacity properly. This would include developing a larger item pool and revalidating the questionnaire with a criterion of at least three items with high loadings in each scale.

As the analyses of health promotion capacity included respondents with missing data on one or more items within a domain, we recognize the risk of bias in the results reported on health promotion capacity, especially for the domains including only 2 items; *students* and *teaching staff*. The majority of respondents had no or only one missing item within a domain, and since we took the mean of responses per school for teachers and school managers separately, the risk of biased responses is considered low.

With an inclusion of 67% of all Danish VET schools (58 of 87 schools), the representativeness is high. However, as the major reason for non-participation (15 schools) was lack of time [34], it is possible that these schools may differ on issues of health promotion capacity and other resources. Moreover, while school managers were highly represented at all 58 included schools, teachers only participated at 38 schools, weakening both the representativeness and power of the results at teacher level. Teachers were further selected by managers, inducing a risk of selection bias, despite measures taken to avoid this. Finally, our non-response analysis confirmed that the included and non-included schools were highly comparable, and our results are overall considered generalizable to the Danish VET schools at the basic program level.

## Conclusions

This prospective study did not find an association between VET schools’ health promotion capacity and student dropout rates during a one-year follow-up period. As both student dropout and unhealthy risk behavior is prominent among VET students, potentially inducing a great risk of inequity in health, we recommend future studies to explore the impact of the schools’ health promotion capacity on student dropout rates with a longer follow-up time, possibly also involving the students’ perspective. Moreover, this study highlights the need for future in-depth research focusing on the content of specific health promotion initiatives that may lower VET school’s student dropout rates. Finally, we call for further development and validation of instruments measuring health promotion capacity in a VET school context and more generally.

## Supplementary information

**Additional file 1. **Factor analyses ^a^ (model fit measures, factor loading, *p*-value) and Cronbach’s alpha tests ^b^ (α) on three different factor structures of health promotion capacity (answered by school managers and teachers).

**Additional file 2.** Number of respondents with missing data (incl. Those who answered “don’t know”) within six health promotion capacity domains.

## Data Availability

The datasets generated on Danish VET school’s student dropout rates during the current study are freely available in the Ministry of Education repository; http://uddannelsesstatistik.dk/pages/erhvervsudd.aspx [30]. Datasets generated and analyzed during the current study are available from the corresponding author on reasonable request.

## Abbreviations

VET: Vocational education and training
CI: Confidence interval
SD: Standard deviations
