# Supplementary material for: Dropout at Danish vocational schools: does the school’s health promotion capacity play a role? A survey- and register-based prospective study
Source: BMC Public Health. 2020 May 26;20:786. doi: 10.1186/s12889-020-08955-4 (PMC7249319; doi:10.1186/s12889-020-08955-4)
Supplement: Supplementary file 1 — Additional file 1. Factor analyses a (model fit measures, factor loading, p-value) and Cronbach’s alpha tests b (α) on three different factor structures of health promotion capacity (answered by school managers and teachers). [file 12889_2020_8955_MOESM1_ESM.docx]

| **Appendix 1**: Factor analyses ^a^ (model fit measures, factor loading, p-value) and Cronbach’s alpha tests ^b^ (α) on three different factor structures of health promotion capacity (answered by school managers and teachers) | | | | | | | | | | | | | |  |  |
| --- | --- | --- | --- | --- | --- | --- | --- | --- | --- | --- | --- | --- | --- | --- | --- |
| **Model A (24 items)** | | | | | **Model B (21 items)** | | | | | **Model C (20 items) – Used for final analysis** | | | | | |
| **Model fit measures: CFI:** 0.828  **TLI:** 0.802  **RMSEA (95% CI):** 0.080 (0.075; 0.085) | | | | | **Model fit measures: CFI:** 0.879  **TLI:** 0.857  **RMSEA (95% CI):** 0.076 (0.070; 0.082) | | | | | **Model fit measures: CFI:** 0,898  **TLI:** 0,874  **RMSEA** (95% CI): 0.073 (0.067; 0079) | | | | | |
| **Domain/item** | **Factor loading** | **p- value** | **α** | **Domain/item** | | **Factor loading** | **p-value** | **α** | **Domain/item** | | **Factor loading** | **p- value** | **α** | |  |
| **Knowledge development**  1. Systematic use of knowledge  2. Cross-sectional collaboration  3. Professional network of employees  4. Influence (teaching staff) | 0.730  0.744  0.685  0.435 | 0.000  0.000  0.000 | 0.76 | **Knowledge development**  1. Systematic use of knowledge  2. Cross-sectional collaboration  3. Professional network of employees  4. Influence (teaching staff) | | 0.730  0.744  0.686  0.434 | 0.000  0.000  0.000 | 0.76 | **Knowledge development**  1. Systematic use of knowledge  2. Cross-sectional collaboration  3. Professional network of employees | | 0.739  0.753  0.703 | 0.000  0.000 | 0.80^c^ | |  |
| **Communication**  1. Daily focus on health promotion  2. Increased focus after VET reform  3. Clear communication to outsiders | 0.794  0.607  0.715 | 0.000  0.000 | 0.72 | **Communication**  1. Daily focus on health promotion  2. Increased focus after VET reform  3. Clear communication to outsiders | | 0.792  0.607  0.715 | 0.000  0.000 | 0.72 | **Communication**  1. Daily focus on health promotion  2. Increased focus after VET reform  3. Clear communication to outsiders | | 0.794  0.607  0.717 | 0.000  0.000 | 0.75^d^ | |  |
| **Resources**  1. Priority  2. Time  3. Physical environment  4. Finances  5. Staff  6. Absence of staff inhibits activities  7. Absence of students inhibits activities  8. External collaborators | 0.620  0.647  0.221  0.446  0.478  0.167  0.076  0.568 | 0.000  0.000  0.000  0.000  0.000  0.16  0.000 | 0.61 | **Resources**  1. Priority  2. Time  3. Finances  4. Staff  5. External collaborators | | 0.637  0.656  0.405  0.441  0.583 | 0.000  0.000  0.000  0.000 | 0.63 | **Resources**  1. Priority  2. Time  3. Finances  4. Staff  5. External collaborators | | 0.656  0.679  0.390  0.424  0.564 | 0.000  0.000  0.000  0.000 | 0.67^e^ | |  |
| **School-based leadership**  1. Health promotion important  2. Common goal setting  3. Leader-based advocacy  4. Support  5. Activities initiated by leaders | 0.795  0.814  0.827  0.767  0.842 | 0.000  0.000  0.000  0.000 | 0.90 | **School-based leadership**  1. Health promotion important  2. Common goal setting  3. Leader-based advocacy  4. Support  5. Activities initiated by leaders | | 0.795  0.814  0.827  0.767  0.842 | 0.000  0.000  0.000  0.000 | 0.90 | **School-based leadership**  1. Health promotion important  2. Common goal setting  3. Leader-based advocacy  4. Support  5. Activities initiated by leaders | | 0.798  0.810  0.828  0.775  0.839 | 0.000  0.000  0.000  0.000 | 0.91^f^ | |  |
| **Teaching staff**  1. Involvement | - | - | - | **Teaching staff**  1. Involvement | | - | - | - | **Teaching staff**  1. Involvement  2. Influence (teaching staff) | | 0.815  0.517 | 0.000 | 0.60^g^ | |  |
| **Students**  1. Involvement  2. Student resources  3. Opportunities to promote own health | 0.665  0.313  0.627 | 0.000  0.000 | 0.57 | **Students**  1. Involvement  2. Student resources  3. Opportunities to promote own health | | 0.667  0.312  0.625 | 0.000  0.000 | 0.57 | **Students**  1. Involvement  2. Opportunities to promote own health | | 0.630  0.564 | 0.000 | 0.55^h^ | |  |
| **^a^** Based on n=528 respondents (excluding those with missing data or had answered “don’t know” to all questions. Missing data on single items are imputed by full information maximum likelihood).  ^b^ Based on n=314-420 respondents depending on domain, as “don’t know” answers are not imputed in the tests; ^c^ n=355, ^d^ n=410, ^e^ n=314, ^f^ n=365, ^g^ n=441, ^h^ n=420. | | | | | | | | | | | | | |  |  |
