# Supplementary material for: Dropout at Danish vocational schools: does the school’s health promotion capacity play a role? A survey- and register-based prospective study
Source: BMC Public Health. 2020 May 26;20:786. doi: 10.1186/s12889-020-08955-4 (PMC7249319; doi:10.1186/s12889-020-08955-4)
Supplement: Supplementary file 2 — Additional file 2. Number of respondents with missing data (incl. Those who answered “don’t know”) within six health promotion capacity domains. [file 12889_2020_8955_MOESM2_ESM.docx]

| **Appendix 2:** Number of respondents with missing data (incl. those who answered “don’t know”) within six health promotion capacity domains. | | | |
| --- | --- | --- | --- |
| **Health promotion capacity domain** | **Teachers**  n (% of n=188) | **School managers**  n (% of n=125) | **Total**  n (% of n=313) |
| **Knowledge development – 3 items** |  |  |  |
| **0 missing ^a^**  **1 missing ^a^**  2 missing  3 missing ^b^ | **110 (58.5)**  **40 (21.3)**  18 (9.6)  20 (10.6) | **105 (84.0)**  **12 (9.6)**  5 (4.0)  3 (2.4) | **215 (68.7)**  **52 (16.6)**  23 (7.3)  23 (7.3) |
| **Communication – 3 items**  **0 missing ^a^**  **1 missing ^a^**  2 missing  3 missing ^b^ | **130 (69.1)**  **36 (19.1)**  12 (6.4)  10 (5.3) | **115 (92.0)**  **8 (6.4)**  2 (1.6)  0 (0.0) | **245 (78.3)**  **44 (14.1)**  14 (4.5)  10 (3.2) |
| **Resources – 5 items**  **0 missing ^a^**  **1 missing ^a^**  2 missing ^a^  3 missing  4 missing  5 missing ^b^ | **89 (47.3)**  **40 (21.3)**  21 (11.2)  17 (9.0)  9 (4.8)  12 (6.4) | **104 (83.2)**  **13 (10.4)**  4 (3.2)  1 (0.8)  0 (0.0)  3 (2.4) | **193 (61.7)**  **53 (16.9)**  25 (8.0)  18 (5.8)  9 (2.9)  15 (4.8) |
| **School-based leadership – 5 items**  **0 missing ^a^**  **1 missing ^a^**  2 missing ^a^  3 missing  4 missing  5 missing ^b^ | **117 (62.2)**  **16 (8.5)**  20 (10.6)  14 (7.4)  5 (2.7)  15 (8.0) | **109 (87.2)**  **5 (4.0)**  4 (3.2)  5 (4.0)  0 (0.0)  2 (1.6) | **226 (72.2)**  **21 (6.7)**  24 (7.7)  19 (6.1)  5 (1.6)  18 (5.8) |
| **Teaching staff – 2 items**  **0 missing ^a^**  1 missing  2 missing ^b^ | **145 (77.1)**  34 (18.1)  9 (4.8) | **119 (95.2)**  6 (4.8)  0 (0.0) | **264 (84.3)**  40 (12.8)  9 (2.9) |
| **Students – 2 items**  **0 missing ^a^**  1 missing  2 missing ^b^ | **139 (73.9)**  36 (19.1)  13 (6.9) | **118 (94.4)**  5 (4.0)  2 (1.6) | **257 (82.1)**  41 (13.1)  15 (4.8) |
| ^a^ The number of missing items is less than 1/3 of items of the domain, i.e. respondents have provided data on more than 2/3 of items of the domain. ^b^ Respondents with missing data on all items is not considered to influence the validity of the domain or factor structure | | | |
